# Pandemic Influenza (H1N1) 2009 Pneumonia: CURB-65 Score for Predicting Severity and Nasopharyngeal Sampling for Diagnosis Are Unreliable

**DOI:** 10.1371/journal.pone.0012849

**Published:** 2010-09-21

**Authors:** Siobhain Mulrennan, Simone Sara Tempone, Ivan Thian Wai Ling, Simon Hedley Williams, Gek-Choo Gan, Ronan John Murray, David John Speers

**Affiliations:** 1 Department of Respiratory Medicine, Sir Charles Gairdner Hospital, Nedlands, Western Australia, Australia; 2 Department of Microbiology, PathWest Laboratory Medicine Western Australia, Nedlands, Western Australia, Australia; 3 Department of Radiology, Sir Charles Gairdner Hospital, Nedlands, Western Australia, Australia; 4 Department of Infectious Diseases, Sir Charles Gairdner Hospital, Nedlands, Western Australia, Australia; 5 Department of Medicine and Pharmacology, University of Western Australia, Crawley, Western Australia, Australia; University of Cape Town, South Africa

## Abstract

**Background:**

From the first case reports of pandemic influenza (H1N1) 2009 it was clear that a significant proportion of infected individuals suffered a primary viral pneumonia. The objective of this study was twofold; to assess the utility of the CURB-65 community acquired pneumonia (CAP) severity index in predicting pneumonia severity and ICU admission, and to assess the relative sensitivity of nasopharyngeal versus lower respiratory tract sampling for the detection of pandemic influenza (H1N1) CAP.

**Methods:**

A retrospective cohort study of 70 patients hospitalised for pandemic influenza (H1N1) 2009 in an adult tertiary referral hospital. Characteristics evaluated included age, pregnancy status, sex, respiratory signs and symptoms, smoking and alcohol history, CURB-65 score, co-morbidities, disabling sequelae, length of stay and in-hospital mortality outcomes. Laboratory features evaluated included lymphocyte count, C-reactive protein (CRP), nasopharyngeal and lower respiratory tract pandemic influenza (H1N1) 2009 PCR results.

**Results:**

Patients with pandemic (H1N1) 2009 influenza CAP differed significantly from those without pneumonia regarding length of stay, need for ICU admission, CRP and the likelihood of disabling sequelae. The CURB-65 score did not predict CAP severity or the need for ICU admission (only 2/11 patients admitted to ICU had CURB-65 scores of 2 or 3). Nasopharyngeal specimens for PCR were only 62.9% sensitive in CAP patients compared to 97.8% sensitivity for lower respiratory tract specimens.

**Conclusions:**

The CURB-65 score does not predict severe pandemic influenza (H1N1) 2009 CAP or need for ICU admission. Lower respiratory tract specimens should be collected when pandemic (H1N1) 2009 influenza CAP is suspected.

## Introduction

April 2009 heralded the advent of the first influenza pandemic for 41 years. Cases of the novel pandemic influenza (H1N1) 2009 were identified in Mexico and Southern California and reports of the clinical outcomes of hospitalised patients soon followed [1], [2]. Data from Mexico [1] demonstrated that pandemic (H1N1) 2009 could cause severe respiratory illness in previously healthy young to middle aged people and pregnant females, as well as those with underlying medical conditions. Pandemic influenza (H1N1) 2009 pneumonia was characterised by fever cough, dyspnoea or respiratory distress and patchy alveolar infiltrates. Since then reports of the clinical features have described either mild to moderate pneumonia [3] or severe pneumonia [4], [5]. Diagnosis of pandemic influenza (H1N1) 2009 infection in these studies was confirmed with reverse transcriptase PCR (RT-PCR) testing, mostly on nasopharyngeal samples, and did not assess the relative sensitivity between upper and lower respiratory tract sampling. Moreover, the utility of community acquired pneumonia (CAP) severity indices for pandemic influenza (H1N1) 2009 pneumonia, such as the CURB-65 score, have not been assessed.

When pandemic influenza (H1N1) 2009 first appeared in Australia in May 2009, it quickly became the dominant circulating influenza strain through the Australian winter and as of October 16, over 37 000 laboratory-confirmed cases had been recorded, resulting in nearly 5 000 hospitalizations and 186 deaths [6]. We describe the clinical and laboratory features of patients diagnosed with pandemic influenza (H1N1) 2009 at Sir Charles Gairdner Hospital, a 650 bed tertiary referral hospital in Perth, Western Australia over a one-month period focussing on the characteristics of individuals with and without CAP. Specifically, we aimed to clarify the utility of the CURB-65 score in predicting pneumonia severity and ICU admission and to assess the relative sensitivity of upper and lower respiratory tract specimens for PCR diagnosis of pandemic (H1N1) 2009 in the setting of pneumonia.

## Methods

We conducted a retrospective review of patients with pandemic influenza (H1N1) 2009 attending our hospital over a one-month period during the winter peak of influenza activity between July 6th and August 6th 2009. A suspected pandemic influenza (H1N1) 2009 case was defined as fever, or history of fever, with acute respiratory symptoms of cough and/or sore throat. All suspected cases were either isolated or cohorted, and had nose and throat swabs collected together with sputum or bronchoscopy samples if these were clinically indicated. Suspected and confirmed cases were initially treated with oseltamivir 75 mg twice daily and, if deemed appropriate, antibacterial therapy. Pandemic influenza (H1N1) 2009 pneumonia (with/without bacterial co-infection and from hereon referred to as the pneumonia group) was defined as symptoms and/or signs of lower respiratory tract infection together with new pulmonary infiltrates on imaging and a positive real-time RT-PCR (rRT-PCR) on a lower respiratory tract sample. Two of the 70 patients did not receive chest imaging and these, along with individuals with chronic lung disease without new infiltrates, were categorized as non-pneumonia pandemic influenza (H1N1) 2009.

Clinical information was obtained by chart review. The data collected included sex, age, pregnancy status, comorbidities, smoking and alcohol consumption, respiratory signs and symptoms, days of symptoms prior to hospitalisation, CURB-65 score at hospital presentation, need for ICU admission, length of hospital stay, and subsequent disability and in-hospital death. This study was considered to be audit activity according to institutional and National Health and Medical Research Council criteria [7], [8], and therefore formal ethics committee approval and informed consent from the patients was not required. Audit approval was sought and granted.

### Laboratory testing

All samples were processed for pandemic influenza (H1N1) 2009 using routine procedures by PathWest Laboratory Medicine WA, Queen Elizabeth II Medical Centre as previously described [9], [10]. Upper respiratory tract specimens included nose and throat swabs collected using either plastic-shafted dacron swabs placed into viral transport medium (VTM) or dry cotton-tipped wire swabs that were vortexed in VTM in the laboratory. Lower respiratory tract specimens included expectorated sputum, sputum aspirated via an endotracheal tube, and bronchoalveolar lavage fluid. RNA extraction from nasopharyngeal swabs was performed as described elsewhere [10]. Viscose samples, including those from the lower respiratory tract, were extracted using the QIAamp Viral RNA Mini kit (QIAGEN, Germany) according to manufacturer specifications.

Amplification was performed by rRT-PCR directed to specific targets in the matrix genes of influenza A and B, and the haemagglutinin genes of H1 (seasonal), H1 (pandemic) and H3 of influenza A [9], [10]. An internal PCR inhibitor control was included for all samples. Cycling was performed using RotogeneQ real-time thermocyclers (QIAGEN, Germany). The rRT-PCR assays were performed either in single target reactions or duplex reactions. Assay sensitivity and specificity was comparable between both methods and is described in detail elsewhere [10]. The cycling threshold (CT) for the PCR reaction, a measure of the strength of the PCR signal that is inversely proportional to the amount of target genetic material present in the specimen, was recorded for each sample. Pneumonia patients were recommended to undergo repeat nasopharyngeal and/or sputum sampling for rRT-PCR every three days during their admission until clinically improved.

Bacterial co-infection was diagnosed if the patient returned a positive culture result for pathologic bacteria from a sterile site (eg blood) and/or lower respiratory tract specimens, or seroconversion to atypical bacterial pathogens (eg Mycoplasma pneumoniae, Chlamydophila pneumoniae, Legionella spp). The lymphocyte count and C-reactive protein (CRP) level were also recorded, as lymphopoenia and raised CRP has been previously reported with pandemic influenza (H1N1) 2009 [3].

### Statistics

We performed statistical analysis using GraphPad Prism software, version 5.02 (GraphPad Software, Inc., San Diego California USA). We report data for continuous variables as medians and used the Mann-Whitney test. Categorical variables were analysed using the Fisher's exact test. Two-tail P values <0.05 were considered significant.

## Results

All patients meeting the case definition for suspected pandemic influenza (H1N1) 2009 underwent nasopharyngeal swabbing for rRT-PCR testing together with sputum or bronchoscopy samples if available, then commenced oseltamivir. The case definition had a pre-test predictive value for a positive rRT-PCR result of approximately 50% (data not shown) and 70 admitted patients (35 male) were confirmed as having pandemic influenza (H1N1) 2009 between July 6^th^ and August 6^th^ 2009. Greater than 50% of patients were aged between 18 and 40 years and 83% were aged between 18 and 60 years. There were eight immunosuppressed patients; four had received solid organ transplants, two were receiving cytotoxic chemotherapy, one had Addison's disease and one had systemic lupus erythematosus.

The baseline characteristics of 35 patients with pandemic influenza (H1N1) 2009 pneumonia were compared to the 35 non-pneumonia cases (Table 1).

**Table 1:** Baseline characteristics of patients with or without pandemic influenza (H1N1) 2009 pneumonia.

Characteristics		Value (%)		p-Value
	All patients	Pneumonia	Non-pneumonia	
**M/F**	35/35 (50)	20/15 (57)	15/20 (43)	0.3
**Median (range) age (years)**	38 (18–76)	38 (21–70)	38 (18–76)	1.0
**Age Group**				
18 to 40	38 (54)	19 (54)	19 (54)	
40 to 60	22 (31)	12 (34)	10 (29)	
60+	10 (14)	4 (11)	6 (17)	
Median (range) days of symptomsprior to presentation	4 (1–21)	5 (1–14)	3 (1–21)	0.4
Median (range) length of stay (days)	4 (0–74)	6 (1–74)	3 (0–23)	**0.002**
**Symptom or outcome**				
Cough	63 (90)	31 (89)	32 (91)	1.0
Fever/rigors	34 (49)	16 (46)	18 (51)	0.8
Dyspnoea	31 (44)	19 (54)	12 (34)	0.1
Sputum production	30 (43)	13 (37)	17 (49)	0.5
Myalgia/arthralgia	18 (26)	9 (26)	9 (26)	1.0
Sore throat	17 (24)	5 (14)	12 (34)	0.1
Wheeze	14 (20)	8 (23)	6 (17)	0.8
Coryza	7 (10)	4 (11)	3 (9)	1.0
Headachce	6 (9)	1 (3)	5 (14)	0.1
Admitted to ICU	11 (16)	11 (31)	0	**<0.001**
Bacterial co-infection	9 (13)	5 (14)	4 (11)	1.0
Disabling sequelae	9 (13)	9 (26)	0	**0.002**
Death	2 (3)	2 (6)	0	0.5
**Comobidities**				
Asthma	19 (27)	12 (34)	7 (20)	0.3
Diabetes	12 (17)	6 (17)	6 (17)	1.0
COPD/emphysema	6 (9)	1 (3)	5 (14)	0.1
Cystic fibrosis	5 (7)	2 (6)	3 (9)	1.0
Pregnancy	5 (7)	4 (11)	1 (3)	0.4
Immunosuppressed	8 (8)	3 (9)	5 (14)	0.7
**Smoking history**				
Current smoker	24 (34)	13 (37)	11 (31)	0.8
Ex-smoker	8 (11)	6 (17)	2 (6)	0.3
Non-smoker	26 (37)	11 (31)	15 (43)	0.5
Not recorded	12 (17)	5 (14)	7 (20)	0.8
**Alcohol consumption**				
Yes	18 (26)	7 (20)	11 (31)	0.3
No	19 (27)	10 (29)	9 (26)	1.0
Not recorded	33 (47)	18 (51)	15 (43)	0.6
**CURB score**				
0		24 (69)		
1		7 (20)		
2		3 (9)		
3		1 (3)		
**Laboratory data**				
Median (range) lymphocyte nadir(x10^9^/L)	0.73(0.00–2.54)	0.68(0.00–2.15)	0.83(0.30–2.54)	0.1
Median (range) CRP peak (mg/L)	72 (5.6–440)	97 (16–390)	51 (5.6–440)	**0.048**

Eleven pneumonia patients required ICU admission; ten required intubation and ventilation, one was observed in ICU overnight, three required extracorporeal membrane oxygenation (ECMO) and two died. The significant differences between the non-pneumonia and pneumonia pandemic influenza (H1N1) 2009 cases were the increased frequency of ICU care, the prolonged length of hospital stay, more disability following infection and a higher peak CRP level (reference range <5 mg/L).

When the CURB-65 score was applied to the pneumonia patients 31 of the 35 (88%) patients had a CURB-65 score of only 0 or 1. At the time of ICU admission only one of the 11 patients had a CURB-65 score of 2 and one patient had a score of 3. The remaining nine (82%) ICU admissions had a score of 0 (n = 6) or 1 (n = 3). The 2 patients who died had a CURB-65 score of 1 and 3 and 2 patients with a CURB-65 score of 2 did not require ICU admission.

There was no difference in the rate of bacterial co-infection in patients with and without pneumonia (5/35 and 4/35 respectively, p = 1.0) indicating the majority of the pneumonia group had a primary viral pneumonia.

**Figure 1:**
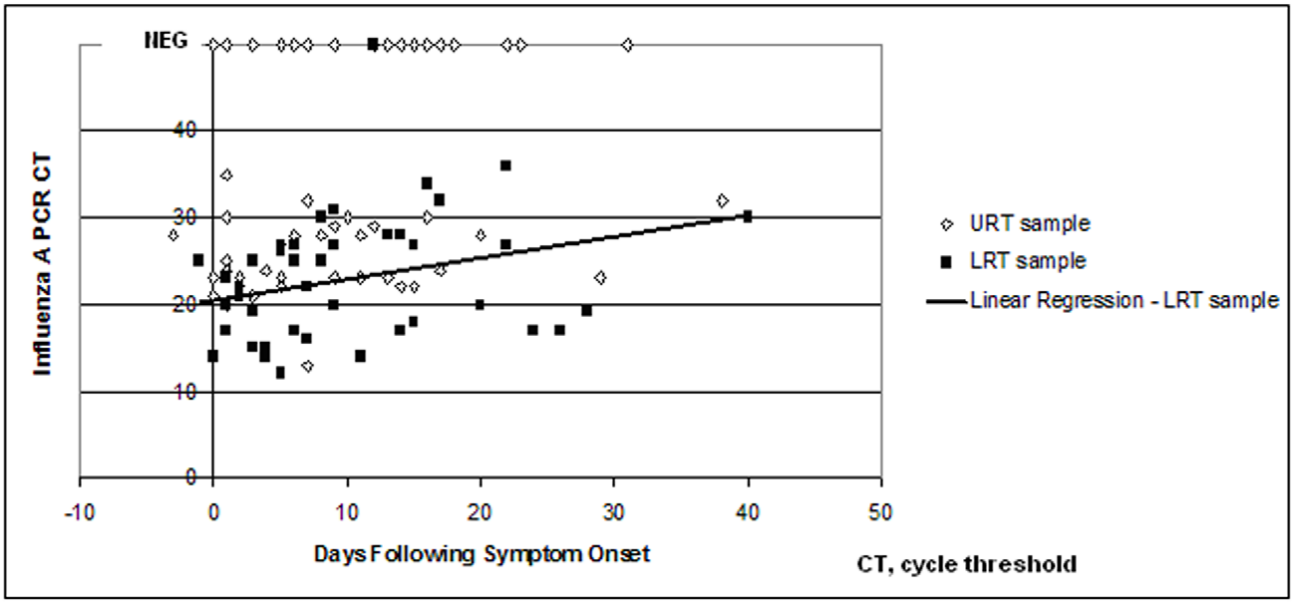
Pandemic influenza (H1N1) 2009 rRT-PCR detections for nasopharyngeal and lower respiratory tract samples from 25 pneumonia patients.

Twenty five pneumonia patients had both nasopharyngeal and lower respiratory tract samples collected. All but one pneumonia patient on one occasion recorded positive lower respiratory tract rRT-PCR results (97.8% sensitivity) but only 62.9% of the nasopharyngeal samples were rRT-PCR positive (fig. 1). Despite the commencement of oseltamivir for all patients on admission, the lower respiratory tract specimens remained positive for many days with only a slow rise in the rRT-PCR CT value (fig. 1).

**Figure 2:**
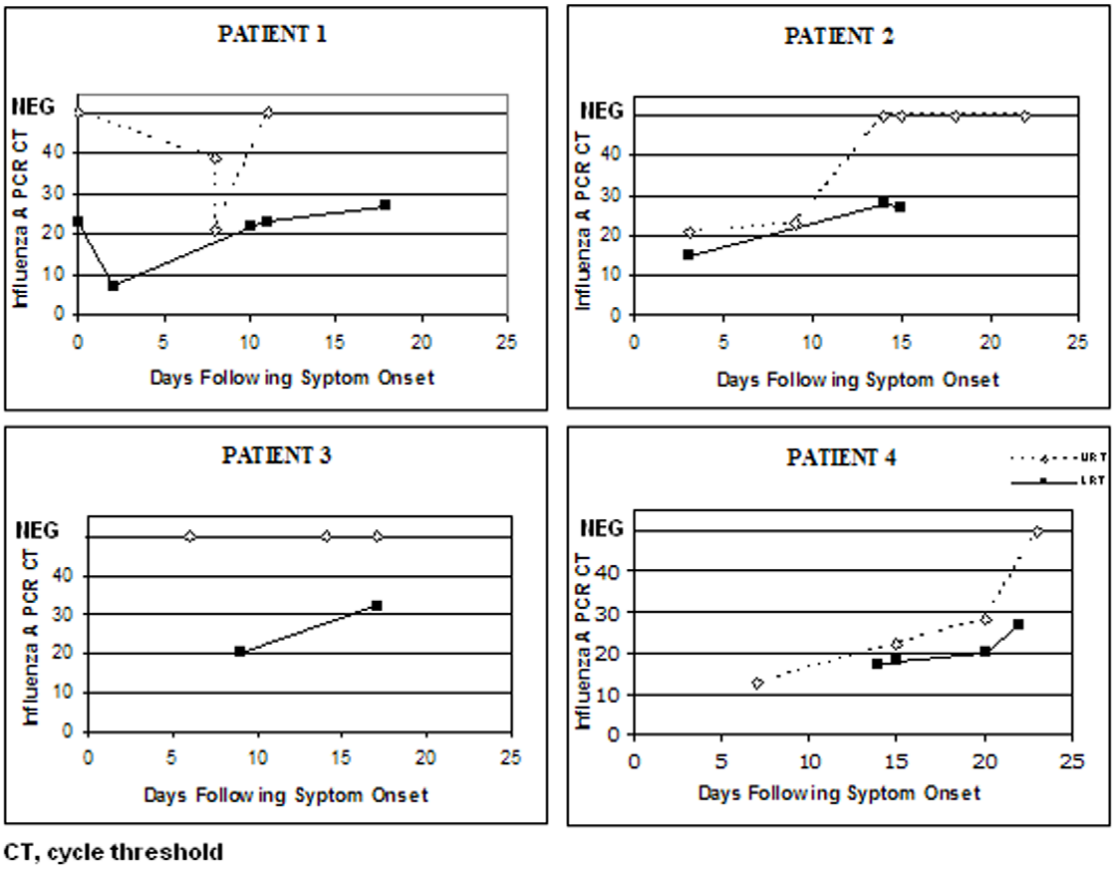
Representative patterns of nasopharyngeal and lower respiratory tract pandemic influenza (H1N1) 2009 rRT-PCR results from four pneumonia patients.

Several patterns of respiratory tract rRT-PCR positivity were revealed by following individual pneumonia patients with repeat upper and lower respiratory tract samples (fig. 2)

## Discussion

Pandemic influenza (H1N1) 2009 can be associated with severe illness and death in previously healthy individuals. The patient characteristics of our cohort mirror those described elsewhere in the literature with the majority of adult patients suffering more severe disease requiring admission aged between 18 and 40 (Table 1). Reported comorbidities included asthma, COPD, diabetes, immunosuppression and pregnancy [3], [5], [11]. As expected, those with pneumonia had a worse clinical outcome with an increase median length of hospital stay, with almost one-third requiring ICU admission, and more disability following their influenza. Only two pneumonia deaths were recorded, which may be a reflection of the intensive supportive care the pneumonia patients received, or the younger age and lack of pre-existing comorbidities in this group compared to the patients usually admitted for CAP following seasonal influenza.

The CURB-65 score, a widely used tool used to enable stratification of CAP patients into mortality risk groups facilitating management including the need for ICU assessment (score 4 or 5) [12], was unhelpful in assessing the severity of influenza pneumonia in our cohort. Buising *et al* [13] found this score predicted mortality well but was only 58% sensitive for predicting ICU admission in their single centre study. In our centre nearly 90% of those with pneumonia or those requiring ICU admission had a CURB-65 score of 0 or 1. According to the CURB-65 results, 89% of our pneumonia cohort and 82% of those admitted to ICU would be categorised as potentially suitable for management as outpatients. Our findings therefore extend those of Buising *et al* [13] that the CURB-65 score, when applied to pandemic influenza (H1N1) 2009, is not suitable for predicting ICU admission.

Rapid detection of pandemic (H1N1) 2009 infection is performed using rRT-PCR testing due to the unreliability of rapid antigen tests [14], [15] and our laboratory used the technique extensively throughout the pandemic [10]. We have demonstrated suboptimal sensitivity (62.9%) of nasopharyngeal sampling in establishing the diagnosis of pandemic influenza (H1N1) 2009 pneumonia (fig. 1). Several large case series have relied on RT-PCR testing of nasopharyngeal sampling for diagnosis [1], [3]. Our findings, and those of others [15], suggest that lower respiratory tract samples, if available, should be obtained when investigating for suspected pandemic influenza (H1N1) 2009 pneumonia. The early experience in Mexico [1] showed that only 18% of patients admitted with pneumonia or influenza-like-illness had positive nasopharyngeal swabs for pandemic influenza A (H1N1) 2009. It is possible that a number of these ‘swab negative’ patients were indeed false negatives, which may have been confirmed with lower respiratory tract sampling.

In a cohort of 426 persons with mostly mild pandemic influenza (H1N1) 2009 infection who underwent regular nasopharyngeal swabbing, the rRT-PCR was found to remain positive for a median of 6 days (range, 1–17) [3]. We also sampled our pandemic influenza (H1N1) 2009 pneumonia patients regularly but from both the upper and lower respiratory tract and also found that the nasopharyngeal swabs often became negative after several days. However, the lower respiratory tract samples remained positive for many days, often well after the nasopharyngeal swabs had become negative, with very little reduction in rRT-PCR signal strength as shown by the slow rise in rRT-PCR CT value (fig. 1,2). Clinicians should be wary of relying on nasopharyngeal swab results if using rRT-PCR negativity as an infection control tool for the release of patients from isolation. There are several possible explanations for the relative lack of sensitivity of nasopharyngeal swabs; the viral load in lower respiratory tract secretions may be greater, the collection of a fluid specimen may preserve the viral RNA better than a swab, or there may have been poor sampling technique for nasopharyngeal swabbing resulting in less respiratory secretion obtained. In support of the latter was the observation that some patients were symptomatic yet nasopharyngeal swab-negative on day 1 only to be swab-positive on day 2. Instructions with step-by-step pictures on how to collect nasopharyngeal swabs were circulated to all clinical staff at the start of the winter season but it was impossible to supervise individual collections.

This study has several limitations. Firstly, it was performed in one adult tertiary hospital such that the results may not be generalised to the pediatric population and other regions of the world. Secondly, the rate of bacterial co-infection in the pneumonia group may have been underestimated. Thirdly, the CURB-65 score is the standard severity index applied to our community acquired pneumonia patients admitted to our hospital. We did not apply other severity indices to our pneumonia cohort, which may have been more accurate than the CURB-65 score. Finally, as we did not perform viral culture on all specimens due to limited testing capacity, we could not confirm that patients with ongoing rRT-PCR positivity were infectious to other patients and staff.

In conclusion, pandemic influenza (H1N1) 2009 pneumonia was associated with prolonged hospital admission, increased ICU admission and increased disability compared to non-pneumonia pandemic influenza (H1N1) 2009 in our hospital. The CURB-65 score did not predict pandemic influenza (H1N1) pneumonia severity or subsequent requirement for ICU admission and the most sensitive rapid diagnostic tool for pandemic influenza (H1N1) 2009 pneumonia was rRT-PCR testing on lower respiratory tract samples, as nasopharyngeal samples were often negative. Prolonged rRT-PCR positivity in lower respiratory tract samples was the norm, despite universal use of oseltamivir during admission.
